# Integration of Single-Photon Emitters in 2D Materials with Plasmonic Waveguides at Room Temperature

**DOI:** 10.3390/nano10091663

**Published:** 2020-08-25

**Authors:** Kwang-Yong Jeong, Seong Won Lee, Jae-Hyuck Choi, Jae-Pil So, Hong-Gyu Park

**Affiliations:** 1 Department of Physics, Korea University, Seoul 02841, Korea; kyjeong83@gmail.com (K.-Y.J.); atoum00712@gmail.com (S.W.L.); gujh87@gmail.com (J.-H.C.); thwovlfwkd@gmail.com (J.-P.S.); 2 KU-KIST Graduate School of Converging Science and Technology, Korea University, Seoul 02841, Korea; 3 Center for Molecular Spectroscopy and Dynamics, Institute for Basic Science, Seoul 02841, Korea

**Keywords:** single-photon emitter, plasmonic waveguide, silver nanowire, h-BN defect, quantum plasmonics

## Abstract

Efficient integration of a single-photon emitter with an optical waveguide is essential for quantum integrated circuits. In this study, we integrated a single-photon emitter in a hexagonal boron nitride (h-BN) flake with a Ag plasmonic waveguide and measured its optical properties at room temperature. First, we performed numerical simulations to calculate the efficiency of light coupling from the emitter to the Ag plasmonic waveguide, depending on the position and polarization of the emitter. In the experiment, we placed a Ag nanowire, which acted as the plasmonic waveguide, near the defect of the h-BN, which acted as the single-photon emitter. The position and direction of the nanowire were precisely controlled using a stamping method. Our time-resolved photoluminescence measurement showed that the single-photon emission from the h-BN flake was enhanced to almost twice the intensity as a result of the coupling with the Ag nanowire. We expect these results to pave the way for the practical implementation of on-chip nanoscale quantum plasmonic integrated circuits.

## 1. Introduction

Efficient coupling of a single-photon emitter with an optical waveguide is essential for implementing a quantum photonic integrated circuit [1]. In particular, when the single-photon emitter is coupled to a plasmonic waveguide, the local density of states (LDOS) of the emitter is increased, and the photon emission is enhanced [2,3,4,5,6,7,8]. This feature was observed by integrating single-photon emitters with randomly dispersed metal nanowires on a substrate [9,10,11,12]. However, as the optical properties of the emitter strongly depend on its distance and direction relative to the metal nanowire, it is necessary to precisely place the emitter and nanowire in the desired positions in order to yield a large photon enhancement and a high coupling efficiency.

Recently, the positions of metal nanowires and metal nanoparticles have been controlled by using an atomic force microscopy (AFM) tip to couple them with single-photon emitters such as nanodiamonds containing color centers [13,14,15], quantum dots [16], and defects in hexagonal boron nitride (h-BN) [17]. Single-photon emitters were also reported to have been coupled with surface-plasmon-polariton (SPP) waveguides formed on the metal surface by electron-beam lithography or plasmonic V-grooves fabricated by focused ion-beam (FIB) milling [7,8,9,10,11,12,13,14,15,16,18,19,20]. However, alignment of the plasmonic waveguide at an accurate angle is necessary to investigate the polarization dependence of the single-photon emitter on the efficiency of coupling with the plasmonic waveguide, which has yet to be demonstrated.

The N_B_V_N_ defect in h-BN is well known for its ability to form a promising single-photon source at room temperature by exhibiting ultrabright light emission with linear polarization and a narrow linewidth [21,22,23,24]. Optically detected magnetic resonance (ODMR) was also reported to originate from the h-BN defect [25]. In this study, we demonstrate efficient coupling between the single-photon emitter in h-BN and a plasmonic waveguide in the form of a Ag nanowire. Numerical simulation shows that the efficiency of light coupling between the emitter and the Ag nanowire depends on the position and polarization of the emitter. To precisely control the position and the direction of the nanowire in the experiment, we used a poly(dimethylsiloxane) (PDMS)/poly(propylene carbonate) (PPC) stamping method. The photoluminescence (PL) measurements before and after the coupling of the emitter with the Ag nanowire at room temperature verify that the Ag nanowire enhances the single-photon emission. The propagation of single photons along the plasmonic waveguide is also demonstrated.

## 2. Materials and Methods

### 2.1. Sample Preparation

h-BN flakes (model: BLK-h-BN solution (2D semiconductors, Scottsdale, AZ, USA)) in solution were dispersed onto a SiO_2_ substrate. The sample was annealed at 850 °C for 30 min at 1 Torr under Ar at a flow rate of 100 sccm to prevent oxidation and increase the emitter density. The PDMS surface was exposed to O_2_ plasma with a power of 30 W and a flow rate of 40 sccm. The PPC was spin-coated onto the PDMS at 1000 rpm for 30 s and baked at 60 °C for 3 min. The Ag nanowires dispersed on the PPC were transferred onto the h-BN flakes using the PDMS/PPC method, where the nanowire’s axis was perpendicular to the polarization direction of the emitters. The PDMS and PPC layers were then separated by heating at 140 °C for 20 min. Then, the samples were immersed in acetone for 10 min to completely remove the PPC from the SiO_2_ substrate.

### 2.2. Numerical Simulations

A three-dimensional (3D) finite-difference time-domain (FDTD) method was used for numerical modeling. The simulation domain was divided by a 2.5 nm mesh, and the perfectly matched layer (PML) was employed as the boundary condition. The refractive index and extinction coefficient of Ag were set at 0.266 and 4.05, respectively. The refractive index and extinction coefficient of Au were set at 0.162 and 2.95, respectively. The thickness of the SiO_2_ substrate was 280 nm. The refractive indices of Si and SiO_2_ were 3.93 and 1.46, respectively, at a wavelength of 600 nm. Then, an electric dipole with a wavelength of 600 nm was introduced as the single-photon emitter. The power emitted via various channels was calculated by normalizing it with the power of the dipole on the SiO_2_ substrate. We varied the distance between the dipole and the center of the nanowire from 50 to 200 nm, and we varied the angle between the polarization direction of the dipole and the nanowire axis from 0 to 90°.

### 2.3. Experimental Setup

The optical properties of the single-photon emission were measured using a home-built confocal microscope setup consisting of a system of two sets of galvanometers to control the excitation and emission individually. Pulsed (SuperK EXTREME (NKT Photonics, Birkeød, Denmark)) or continuous-wave (CW) lasers (OBIS 532 nm LS (Coherent Inc., Santa Clara, CA, USA)) with a wavelength of 532 nm were used as the pumping source. Emission from the h-BN was collected by a 100× objective lens that had a numerical aperture of 0.90 and was coupled to the single-mode fiber to send the PL to either the Avalanche photodiode (APD) or the spectrometer (Acton SP2500 (Teledyne Princeton Instruments, Trenton, NJ, USA)). To measure the second-order correlation, we used a Hanbury Brown and Twiss (HBT) interferometer setup composed of two APDs and a time-correlated single-photon counting module (Picoharp 300 (PicoQuant, Berlin, Germany)). The emission from the sample was divided into two APDs using a 50:50 fiber beam splitter. Polarization measurements were conducted by placing a linear polarizer in front of the fiber coupler.

## 3. Results

**Figure 1:**
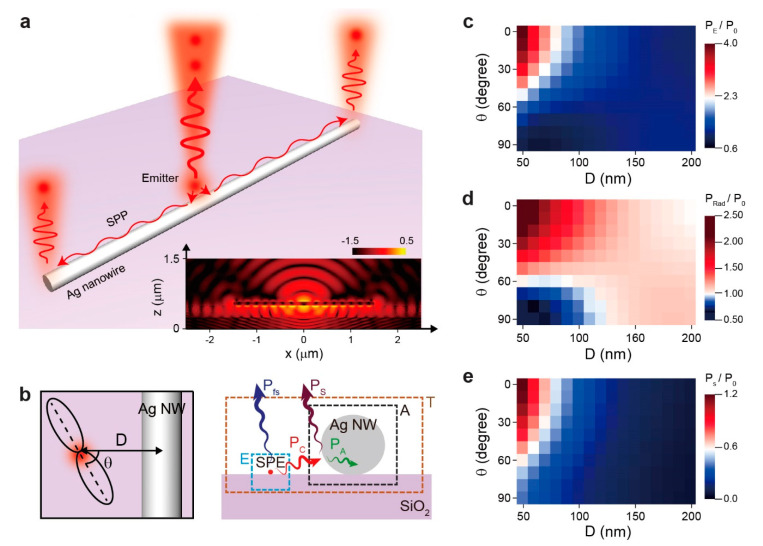
Single-photon emitter coupled to a Ag nanowire and simulation results. (**a**) Schematic illustration. The inset shows a cross-sectional view of the calculated electrical field intensity distribution. (**b**) The schematic on the left shows the distance (D) between the emitter, the Ag nanowire, and the polarization direction (θ) of the emitter. The schematic on the right shows the closed surfaces used to calculate the power emitted via various channels. P_E_, total power from the dipole emitter; P_Rad_, total radiative power; P_fs_, direct radiative power from emitter to free space; P_c_, power transferred to nanowire; P_A_, nonradiative power; P_S_, power scattered by nanowire. (**c**–**e**) Calculated 2D maps of the total power enhancement (**c**), radiative power enhancement (**d**), and scattered power enhancement (**e**), as functions of D and θ. The θ varies from 0 (normal to the nanowire) to 90° (parallel to the nanowire). D varies from 50 to 200 nm.

Figure 1a schematizes the coupling of single photons emitted from a defect in h-BN to the Ag nanowire. The photons propagate along the nanowire surface as single plasmons and are converted to single photons again at the end of the nanowire. While propagating along the Ag nanowire, certain photons are converted to surface plasmons, whereas other photons are absorbed by the metal. This was simulated using our home-built 3D FDTD software. In our simulation, a Ag nanowire with a length of 3 μm and a diameter of 100 nm was placed on the SiO_2_ substrate. An electric dipole with a wavelength of 600 nm was located on the substrate, whereas the distance (D) between the nanowire and the dipole and the polarization direction (θ) of the dipole were varied (Figure 1b, left). We first calculated the electric field intensity distribution when D = 105 nm and θ = 0° (inset of Figure 1a). Light scattering is observed at both ends of the nanowire, whereas the direct emission from the dipole into the air occurs in the middle of the nanowire.

To investigate the effects of D and θ on the coupling efficiency, we calculated the power emission via various channels as functions of D and θ. We considered all possible channels in terms of the following parameters: total power from the dipole emitter (P_E_), total radiative power (P_Rad_), direct radiative power from the emitter to the free space (P_fs_), power from the emitter to the nanowire (P_c_), total nonradiative power (P_A_), and power scattered by the nanowire (P_S_). As shown in the panel on the right in Figure 1b, P_E_, P_Rad_, and P_A_ were calculated by integrating the Poynting vectors over the closed surface E (surrounding the dipole), the closed surface T (surrounding the dipole and nanowire), and the closed surface A (surrounding only the nanowire), respectively. P_S_ was calculated by integrating the Poynting vectors of the scattered fields over the closed surface A, and P_C_ was the sum of P_S_ and P_A_ [26,27].

Figure 1c–e shows P_E_, P_Rad_, and P_S_ normalized by the total emitted power without the Ag nanowire, P_0_, when θ varies from 0 (normal to the nanowire) to 90° (parallel to the nanowire), and D varies from 50 (the radius of the nanowire) to 200 nm. As the dipole emitter approaches the Ag nanowire and the polarization direction of the dipole is normal to the axis of the nanowire, the dipole is efficiently coupled to the Ag nanowire with an increased total power enhancement (Figure 1c). When D is 50 nm and θ is 0°, the total power enhancement is 3.97. In addition, the radiative power enhancement increases as D and θ become smaller (Figure 1d). When D is 50 nm and θ is 0°, the radiative power enhancement is 2.40. Because of the power absorbed by the metal, the radiative power enhancement is less than the total power enhancement. Furthermore, it is important to enhance the radiative power to greater than 1 to obtain a strong single-photon emission. Even if the Ag nanowire is placed near the emitter (D = 50 nm), θ should be smaller than 60° to achieve this objective (i.e., for the radiative power enhancement to be greater than 1). When θ is 0° (normal to the nanowire), the radiative power enhancement is greater than 1 if the D is smaller than 200 nm. This result indicates that both D and θ affect the coupling of single photons to the Ag nanowire.

**Figure 2:**
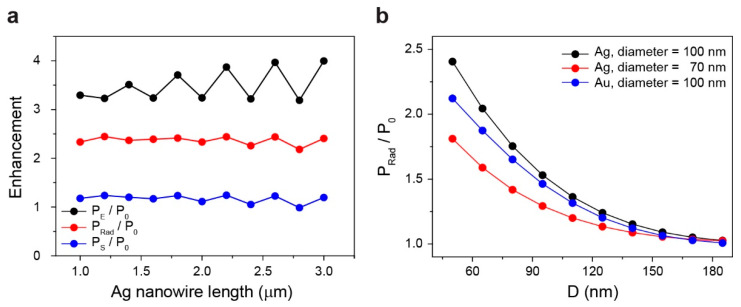
Calculated power enhancements. (**a**) Total power enhancement (black), radiative power enhancement (red), and scattered power enhancement (blue) calculated as a function of the Ag nanowire length when the diameter of nanowire = 100 nm, D = 50 nm, and θ = 0°. (**b**) Radiative power enhancement calculated as a function of D for Ag and Au nanowires. The diameters of 100 nm (black) and 70 nm (red) for the Ag nanowire and the diameter of 100 nm (blue) for the Au nanowire were examined.

Next, we calculated the power enhancements depending on the dimensions and materials of the nanowires (Figure 2). Figure 2a shows the results obtained when only changing the length of the Ag nanowire from 1 to 3 μm, while keeping the nanowire diameter, D, and θ, fixed as 100 nm, 50 nm, and 0°, respectively. The simulation shows that the enhancements of P_E_, P_Rad_, and P_S_ are almost the same even when the nanowire length changes. The power fluctuation of each curve may result from the Fabry–Perot resonance in the Ag nanowire [28]. In addition, we studied the enhancement of P_Rad_ as a function of D for Ag and Au nanowires with different diameters (Figure 2b). Diameters of 100 and 70 nm for the Ag nanowire and a diameter of 100 nm for the Au nanowire were examined, while the other parameters were fixed. In all cases, the radiative power enhancement decreases as the D increases. For a Ag nanowire with a smaller diameter, the electric field intensity of surface plasmons is weaker and the P_Rad_ is smaller for the same D. For the Au nanowire, the power enhancement is also weaker due to the increased optical loss of Au when the other structural parameters are all the same.

**Figure 3:**
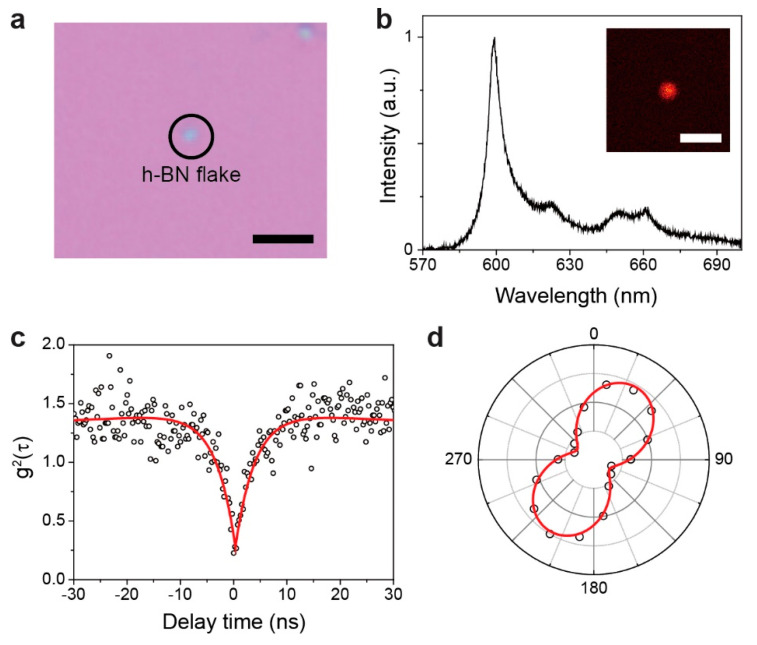
Experimental results. (**a**) Optical microscope image of a hexagonal boron nitride (h-BN) flake on the SiO_2_ substrate. Scale bar, 2 μm. (**b**) Measured spectrum of h-BN by optical pumping at room temperature. The zero phonon line (ZPL) wavelength is 600 nm. The inset shows the confocal photoluminescence (PL) image of single-photon emission. Scale bar, 1 μm. (**c**) Measured second-order correlation function (g^2^(τ)) of the single-photon emission in (**b**) (dots). The red line indicates the fitting curve using a three-level model equation. The value of g^2^(0) is 0.18. (**d**) Measured polarization of ZPL. The polarization visibility is 64%.

To verify these theoretical results, we conducted a coupling experiment between a single-photon emitter (h-BN defect) and a Ag nanowire. First, h-BN flakes were dispersed on a SiO_2_ substrate with markers for alignment (Figure 3a). The optical properties of h-BN defects were measured using a confocal scanning fluorescence microscope setup with a 532 nm CW pump laser. The emitted photons were collected by a 100× microscope objective lens with a numerical aperture of 0.9 that was coupled to a single-mode fiber to send the photons to the APD or spectrometer. Confocal fluorescence scanning of the sample revealed bright and localized spots (inset of Figure 3b). The spectrum of the bright spot exhibits a sharp zero phonon line (ZPL) peak at a wavelength of 600 nm and with a linewidth of 7 nm (Figure 3b). We also measured the second-order autocorrelation (g^2^(τ)) of the photon emission using a Hanbury Brown and Twiss interferometer setup (Figure 3c). The measured g^2^(τ) data were fitted using a three-level model equation:

(1) $$g^{2}(\tau )=1-(ae^{-\frac{\left|\tau \right|}{\tau_{1}}}+\left(a-1)e^{\frac{\left|\tau \right|}{\tau_{2}}})\right)$$

where the parameters τ_1_ and τ_2_ represent the radiative transition lifetime and the metastable state lifetime, respectively, and *a* is a bunching factor. The measured g^2^(0) value was 0.18, exhibiting a clear characteristic of single-photon emission. Additionally, we measured the polarization of the ZPL wavelength. The photon emission is linearly polarized with a polarization visibility of 0.64 (Figure 3d).

**Figure 4:**
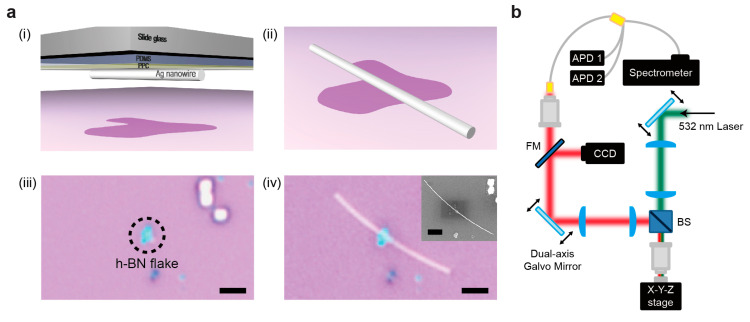
Positioning the Ag nanowire. (**a**) Precise positioning of the Ag nanowire onto the h-BN flake. (i,ii) Schematics of the poly(dimethylsiloxane) (PDMS)/poly(propylene carbonate) (PPC) stamping method. The Ag nanowire dispersed on the PPC/PDMS (i) was aligned and transferred to the h-BN flake (ii). (iii,iv) Optical microscope images of the h-BN flake before (iii) and after (iv) transferring the Ag nanowire. The inset in (iv) shows the scanning electron microscopy (SEM) image of the fabricated sample. Scale bar, 2 μm. (**b**) Schematics of confocal microscope measurement setup with two sets of galvo mirrors. Excitation of samples and PL scanning measurements were performed independently.

Next, the PDMS/PPC stamping method was used to place the Ag nanowire in the desired position near the h-BN flake. The stamping method is comprised of the following steps (additional details are given in Section 2): First, the PPC was spin-coated on the PDMS at 1000 rpm for 30 s and baked at 60 °C for 3 min. After dispersing the Ag nanowires onto the PPC, an isolated single Ag nanowire was selected. This target Ag nanowire was transferred onto a single-photon emitter in the h-BN flake, with the direction of the Ag nanowire perpendicular to the emitter polarization (Figure 4a, (i)). Then, the PDMS film was separated from the PPC film by heating. The remaining PPC on the sample was removed by acetone for 10 min (Figure 4a, (ii)). The optical microscope images of the fabricated samples before and after transferring the Ag nanowire onto the h-BN flake are shown below (Figure 4a, (iii) and (iv)).

The single-photon emissions from a defect in the h-BN, coupled to the Ag nanowire, were measured using a home-built confocal microscope setup that consisted of two sets of galvo mirror scanners to scan the pump position and detect the position independently (Figure 4b). One galvo mirror scanner was used to efficiently pump the h-BN single-photon emitter, whereas the other galvo mirror scanner was used to acquire PL images and position-dependent spectra of the entire sample, including the h-BN and Ag nanowire.

**Figure 5:**
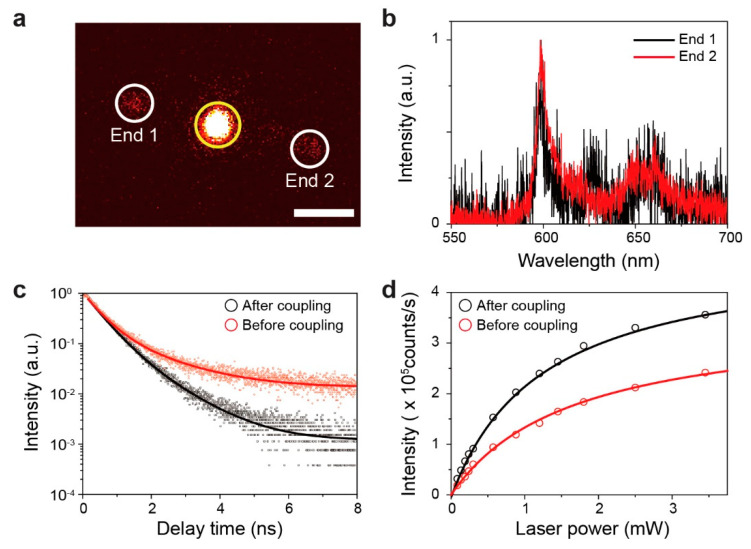
Measurement of Ag nanowire coupled single-photon emission: (**a**) Confocal fluorescence image scan of the single-photon emitter coupled to a Ag nanowire with the excitation laser fixed to the position of the emitter. The yellow and white circles represent the single-photon emitter and two ends of the Ag nanowire, respectively. (**b**) Spectra measured at the ends of the Ag nanowire. These are the same as the spectrum of the h-BN single-photon emitter (Figure 3b). (**c**) Comparison between the time-resolved photoluminescence (TRPL) measurements before and after coupling of the single-photon emitter with a Ag nanowire. The red and black curves were measured before and after coupling, respectively. (**d**) Comparison between the fluorescence saturation curves before and after coupling with a Ag nanowire. The red and black curves were measured before and after coupling, respectively.

Figure 5a shows the confocal PL image scan of the single-photon emission coupled to a Ag nanowire, while the pump laser was focused on the h-BN flake. Strong photon emission from the h-BN defect in the middle of the Ag nanowire (Figure 5a, yellow circle) was coupled to and propagated along the Ag nanowire. As a result, weak photon emission was observed at both ends of the Ag nanowire (Figure 5a, white circles). The spectra measured at both ends of the nanowire were almost the same, showing a well-resolved peak at 600 nm (Figure 5b), and also fairly similar to the spectrum measured before the coupling with the Ag nanowire (Figure 3b). This result indicates that the single-photon emission from the h-BN flake was converted to SPP and propagated along the Ag nanowire.

In addition, we measured the time-resolved PL before and after coupling with the Ag nanowire (Figure 5c). The measured data were fitted by a double exponential decay function. The lifetimes were 2.09 and 1.11 ns before and after the coupling, respectively. The lifetime reduction by the Ag nanowire was attributed to the increased nonradiative and plasmonic decay channels, which led to the increased LDOS of the single-photon emitter. Specifically, the experimentally measured reduction in lifetime by 1.88 times was comparable to the simulation result of the total power enhancement in Figure 1c. In the simulation, this enhancement was obtained when the polarization direction of the single-photon emitter was perpendicular to the Ag nanowire and was positioned at a distance of approximately 100 nm from the center of the Ag nanowire. A comparison between the measured and simulated results indicates that the PDMS/PPC stamping method enabled the Ag nanowire to be placed near the single-photon emitter, with a placement accuracy of less than 100 nm. 

Finally, we measured the fluorescence saturation curves before and after coupling with the Ag nanowire (Figure 5d). The saturation measurements were fitted with the equation:

(2) $$I=I_{\infty }\times P/(P+P_{Sat})$$

where $I_{\infty }$ and $P_{Sat}$ are the saturated emission rate and the excitation power at the saturation, respectively. The saturated emission rates were estimated to be 3.52 × 10^5^ counts/s and 4.90 × 10^5^ counts/s before and after coupling, respectively. This improvement in the saturated emission rate signifies an efficient coupling of single photons with the Ag nanowire.

## 4. Conclusions

In summary, we successfully demonstrated the efficient coupling of h-BN single-photon emitters and Ag nanowire plasmonic waveguides. We analyzed the power enhancement of the emitter as a function of its polarization and position relative to the Ag nanowire by calculating the Poynting vectors through various channels. The simulation showed that the distance between the single-photon emitter and Ag nanowire and the polarization direction of the single-photon emitter affected the coupling efficiency. In the experiment, a Ag nanowire was accurately positioned close to the h-BN single-photon emitter using the PDMS/PPC stamping method. The optical measurement demonstrated the enhanced single-photon emission from h-BN as a result of the emitter coupling with the Ag nanowire. Certain photons were converted to single plasmons, propagated along the surface of the Ag nanowire, and emitted into free space at the ends of the nanowire. In addition, the lifetime of the emitter was determined to be reduced by 1.88 times by the Ag nanowire. A comparison of this result with the simulation result suggested that the h-BN single-photon emitter was positioned at a distance of 100 nm from the Ag nanowire. By fine-tuning the emission wavelength of the single-photon emitter, we expect our results to pave the way toward the practical implementation of quantum plasmonic integrated circuits based on 2D materials and Ag nanowires.
